# Knowledge, attitude and practice of infection prevention and control precautions among laboratory staff: a mixed-methods systematic review

**DOI:** 10.1186/s13756-023-01257-5

**Published:** 2023-06-13

**Authors:** Haifa Aldhamy, Gregory Maniatopoulos, Victoria L. McCune, Ilaf Mansi, Majid Althaqafy, Mark S. Pearce

**Affiliations:** 1 Population Health Sciences Institute, Newcastle University, Baddiley-Clark Building, Newcastle Upon Tyne, NE2 4AX UK; 2 Faculty of Applied Medical Sciences, Qassim University, Qassim, Saudi Arabia; 3 School of Business, University of Leicester, Leicester, UK; 4 Department of Microbiology, South Tees Hospitals NHS Foundation Trust, Middlesbrough, UK; 5 North West Ambulance Service, Lancashire, UK; 6 Infection Prevention and Control, King Abdulaziz Medical City, Jeddah, Saudi Arabia

**Keywords:** Infection prevention and control guidelines, Laboratory safety, Laboratory staff, Knowledge, Attitude, Practice

## Abstract

**Background:**

Clinical laboratories provide diagnostic testing services to support the effective delivery of care in today’s complex healthcare systems. Processing clinical material and the use of chemicals or radiation presents potential hazard to laboratory workers, from both biological and chemical sources. Nevertheless, the laboratory should be a safe workplace if the identification of possible hazards, clear guidelines, safety rules and infection prevention and control (IPC) precautions are applied and followed. The main aim of this systematic review was to identify, critically appraise and synthesise the research evidence to gain a clear explanation of the implementation and knowledge, attitude and practice (KAP) of IPC guidelines among hospital laboratory staff.

**Methods:**

For this systematic review we searched MEDLINE, EMBASE, Scopus and CINAHL (EBSCO), PubMed, grey literature, reference lists and citations for studies published between database inception and November, 2021. All qualitative, quantitative and mixed-methods studies whose aim was to explore risk perception and KAP of IPC guidelines among laboratory staff in any healthcare setting were included, without language or date restrictions. Evidence was narratively synthesised into group of themes. The quality of the evidence was assessed with Joanna Briggs Institutes Critical Appraisal Tools.

**Results:**

After the full-text screening, a total of 34 articles remained and were included in the final review. Thirty papers were considered to be of high quality and the remaining four were considered to be of low quality. The available evidence shows that there was good knowledge, good attitudes and moderate immunisation status, but there was still poor practice of IPC precautions and an inadequate level of training among laboratory workers.

**Conclusion:**

There is a gap among KAP related to the implementation of IPC guidelines, which indicates that laboratory staff may be at high risk of acquiring infections in the workplace. These findings suggest that training (including IPC precautions, safety policies, safety equipment and materials, safety activities, initial biohazard handling, ongoing monitoring and potential exposure) of laboratory staff to increase their knowledge about IPC precautions could improve their use of these precautions.

**Supplementary Information:**

The online version contains supplementary material available at 10.1186/s13756-023-01257-5.

## Background

Clinical laboratories provide diagnostic testing services to support the effective delivery of care in today’s complex healthcare systems [1]. This includes microbiological, serological, biochemical, haematological, cytological and pathological examinations of clinical specimens derived from patients for the purpose of affording information for the diagnosis, treatment or prevention of any disease. Around 70% of clinical decisions are based on information gathered from laboratory testing [2].

Processing clinical material and the use of chemicals or radiation presents potential hazards to laboratory workers, from both biological and chemical sources. Laboratory workers are at risk of exposure to biological hazards through a variety of routes such as: inhalation of aerosols; percutaneous inoculation (needlestick injuries and cuts from contaminated items); contact between contaminated materials (surfaces, hands) and mucous membranes; and ingestion (smoking or eating, aspiration through a pipette) [3]. Laboratory-acquired infection is of particular concern for pathogens such as hepatitis B and C viruses (HBV and HCV), human immunodeficiency viruses (HIV), Middle East Respiratory Syndrome [4] and Severe Acute Respiratory Syndrome Coronavirus 2 [49]. Moreover, the major source of most hepatitis and HIV infections among healthcare professionals is needlestick injury, which can occur during all stages of needle use procedures [5]. Occupational risk and illnesses may occur owing to incorrect practices, failure of the procedures to correctly eliminate or control the risk, poor communication about high-risk patients, lack of compliance, inexperience, ignorance and failure to follow recognised procedures and guidelines. However, the laboratory can be a safe workplace if possible hazards are identified, and clear guidelines, safety rules and infection prevention and control (IPC) precautions are applied and followed [6].

Implementing IPC guidelines provides a practical, evidence-based approach to prevent both patients and health workers from being harmed by avoidable infection and possible hazards. It comprises a set of recommendations created to minimise and prevent harm to healthcare workers and patients induced by exposure to infectious agents [9]. The IPC programmes include standard and transmission-based precautions with which all laboratory and other healthcare workers must familiarise themselves. These precautions involve practices of hand hygiene, the use of personal protective equipment (PPE) (gloves, gowns, masks (N-95, paper, etc.), plastic aprons, face shields and protective eyewear), the safe use and disposal of sharps, routine environmental cleaning and waste management [10].

It should be clarified that in some countries such as the UK, Canada and Germany, IPC guidelines relate more to the clinical work and the prevention of infection transmission on wards only, and the IPC team consists of specialist nursing and medical staff [7]. In the clinical laboratories and other facilities where people may be exposed to biological agents, health and safety guidance is applied [8]. In other countries such as the Kingdom of Saudi Arabia (KSA), United Arab Emirates (UAE), Qatar, and Kuwait the IPC guidelines are applied to the clinical laboratories in addition to the wards, in hospitals and other healthcare settings, and the laboratory staff can be part of the IPC team as well.

To date, there is a lack of evidence about knowledge, attitudes and practice (KAP) with respect to all IPC precautions collectively among laboratory staff. Moreover, no reviews have been conducted on the assessment of KAP of IPC guidelines worldwide. The aim of this systematic review was to identify, critically appraise and synthesise the research evidence related to the implementation and KAP of IPC guidelines among laboratory staff.

The principal objectives of the review were to systematically search for published qualitative, quantitative and mixed-methods studies on the implementation and KAP of IPC guidelines, to synthesise and assess the quality of studies included and to evaluate the existing evidence surrounding the IPC guidelines. Moreover, this review will identify gaps in the data on implementation, adherence and KAP of IPC guidelines among laboratory staff around the world with the aim of identifying priorities for future research.

## Methodology

### Search strategy

A protocol for this systematic review was prepared and followed, and was registered in PROSPERO (CRD42023188876). This systematic review was conducted following the reporting items for systematic reviews and meta-analyses specified by the PRISMA 2020 checklist [52].

A number of electronic databases were searched to locate the relevant studies using a combination of search terms. Databases searched include MEDLINE, EMBASE, Scopus and CINAHL (EBSCO). PubMed and grey literature were also searched. In addition, reference lists and citations of relevant documents identified from databases were searched to locate pertinent studies. No time limit was applied to the search because the aim of this review was to capture all articles existing. The last search of articles was in November 2021.

A search was performed using medical headings that cover the topic of interest, which were then combined using the Boolean operator terms. The search strategy used in MEDLINE was modified for use on other databases searched. The complete search strategy for each database is presented in Tables 1 and 2.

**Table 1:** Search Strategy: Medline and Embase-Ovid

Search term used	
1. Knowledge/ or Knowledge.mp. or Health Knowledge, Attitudes, Practice/ 2. Health perception.mp. 3. Risk perception.mp. 4. 1 OR 2 OR 3 5. Attitude.mp. or “Attitude of Health Personnel”/ or Attitude/ or Attitude to Healt 6. Behaviour.mp. 7. 5 OR 6 8. Clinical practice/Practice Guideline/ or Practice.mp. 9. 4 AND 7 AND 8 10. Implementation.mp. 11. Adherence.mp 12. 10 OR 11 13. Infection control.mp. or Infection Control/ 14. Infection prevention.mp. 15. Universal precautions.mp. or universal precaution/ 16. Infection control/ or standard precautions.mp. 17. policy/ or Policy.mp. 18. Laboratory safety/ safety/ or biosafety/ or occupational safety.mp. 19. Safety precautions.mp. 20. 13 OR 14 OR 15 OR 16 OR 17 OR 18 OR 19 21. 12 AND 20 22. 9 AND 21 23. Healthcare personnel.mp. 24. Laboratory personnel.mp. 25. Medical laboratory personnel.mp. 26. Laboratory specialists.mp. 27. 23 OR 24 OR 25 OR 26 28. 22 AND 27 29. Hospital laboratory/Hospital.mp. or Hospitals/ 30. Secondary care.mp. or Secondary Care/ 31. 29 OR 30 32. 28 AND 31 33. Qualitative research.mp. or Qualitative Research/ 34. Mixed methods.mp. 35. 33 OR 34 37. 32 AND 35	

**Table 2:** Search Strategy: CINAHL

Search term used	
1. knowledge, attitude and practice 2. attitudes or perceptions or opinions or thoughts or feelings or beliefs 3. practice 4. risk perception or perceived risk 5. adherence or compliance 6. implementation 7. 1 OR 2 OR 3 OR 4 OR 5 OR 6 8. infection control or infection prevention or infection control and prevention 9. Laboratory safety 10. 8 OR 9 11. 7 AND 10 12. Laboratory personnel 13. healthcare professionals 14. 12 OR 13 15. 11 AND 14 16. hospital or acute setting or inpatient or ward 17. 15 AND 16 18. qualitative research or qualitative study or qualitative methods or interview 19. quantitative research or quantitative study or quantitative 20. mixed methods or ‘qualitative’ and ‘quantitative’ 21. 18 OR 19 OR 20 22. 17 AND 21	

An EndNote library (version X8) was created for this review and used to download the titles and abstracts after searching each database. This allowed clarification and elimination of any duplicated studies within and between databases.

### Screening

Rayyan Qatar Computing Research Institute (web for systematic reviews) was used to perform the initial title and abstract screening. Then, full texts of the included articles were screened for eligibility by two reviewers independently (HA and IM). Finally, decisions of inclusion/exclusion were made by the reviewers and reasons for exclusion were recorded, and disagreement between reviewers was solved by discussion on each included and excluded paper.

### Inclusion criteria

Studies eligible for inclusion were qualitative, quantitative, mixed-methods research whose authors discussed risk perception and KAP of IPC guidelines among laboratory staff in any healthcare setting including tertiary care settings, primary care settings, long-term care, acute hospital settings or community settings. Studies on awareness or compliance with specific infection control guidelines such as hand hygiene and waste disposal, and studies that covered occupational injuries such as sharp injuries, were also included. Furthermore, studies on laboratory-related infections and safety precautions associated with them and studies focused on different vaccinations required for healthcare workers were included. Also, studies that covered infection control guidelines and safety measure policies and how they change over time in different countries were included. All published literature up to November 2021 was included in this review. There were no restrictions on country of study. However, the included studies had to be published in English.

### Exclusion criteria

Cohort, case-control and randomised controlled trials were excluded from this review. This was because the identified studies did not address the aim of this review and thus did not display any relevant data. For the same reason, studies on the effectiveness of interventions on the KAP of laboratory staff were excluded. Studies were excluded if they were focused on healthcare workers but did not include laboratory staff in the sample as participants, as well as studies on nurses and dental workers only. Studies in which data for laboratory staff could not be separated from the data gathered on other healthcare workers were excluded. Studies on students and university laboratories were excluded. Finally, general discussion papers such as letters, editorials and comments, conference abstracts and poster presentations were also excluded.

### Data extraction

Data on studies that met the inclusion criteria were extracted by one reviewer (HA), and a standardised data extraction form was developed that included the following headings: author/year, main focus of the study, method, country, sample, outcome measures and the study results.

### The quality assessment exercise

Because more than one type of research was included in this review, Joanna Briggs Institutes Critical Appraisal Tools (JBI-CAT) were the relevant option to assess the quality of the included papers. JBI-CAT are designed to be used for multiple study designs with the purpose of assessing the quality of a study methodology and to determine the extent to which the possibility of bias in its design, handling and analysis has been addressed in the study [3]. Two different checklists of JBI-CAT were employed based on the types of included studies (see Additional files 1&2).

### Data synthesis and analysis

A complete reading of the included studies was carried out by HA. Afterwards, the information corresponding to the aim and objective of this review was identified, using the authors’ interpretations and textual quotes (from qualitative studies). Finally, categories and related themes whose origin was the main topic of the study emerged and are shown in the results section.

Owing to the nature of data in this mixed-methods review, and the limited availability of numerical (quantitative) data for applying a meta-analysis approach, a narrative synthesis approach was followed. A narrative synthesis approach is defined as an ‘approach to the systematic review and synthesis of findings from multiple studies that rely primarily on the use of words and text to summarise and explain the findings of the synthesis’ [11]. This approach can be utilised in qualitative, quantitative and mixed-methods studies alike and assists integration of both qualitative and quantitative data to achieve the aim of the review.

## Results

**Fig. 1:**
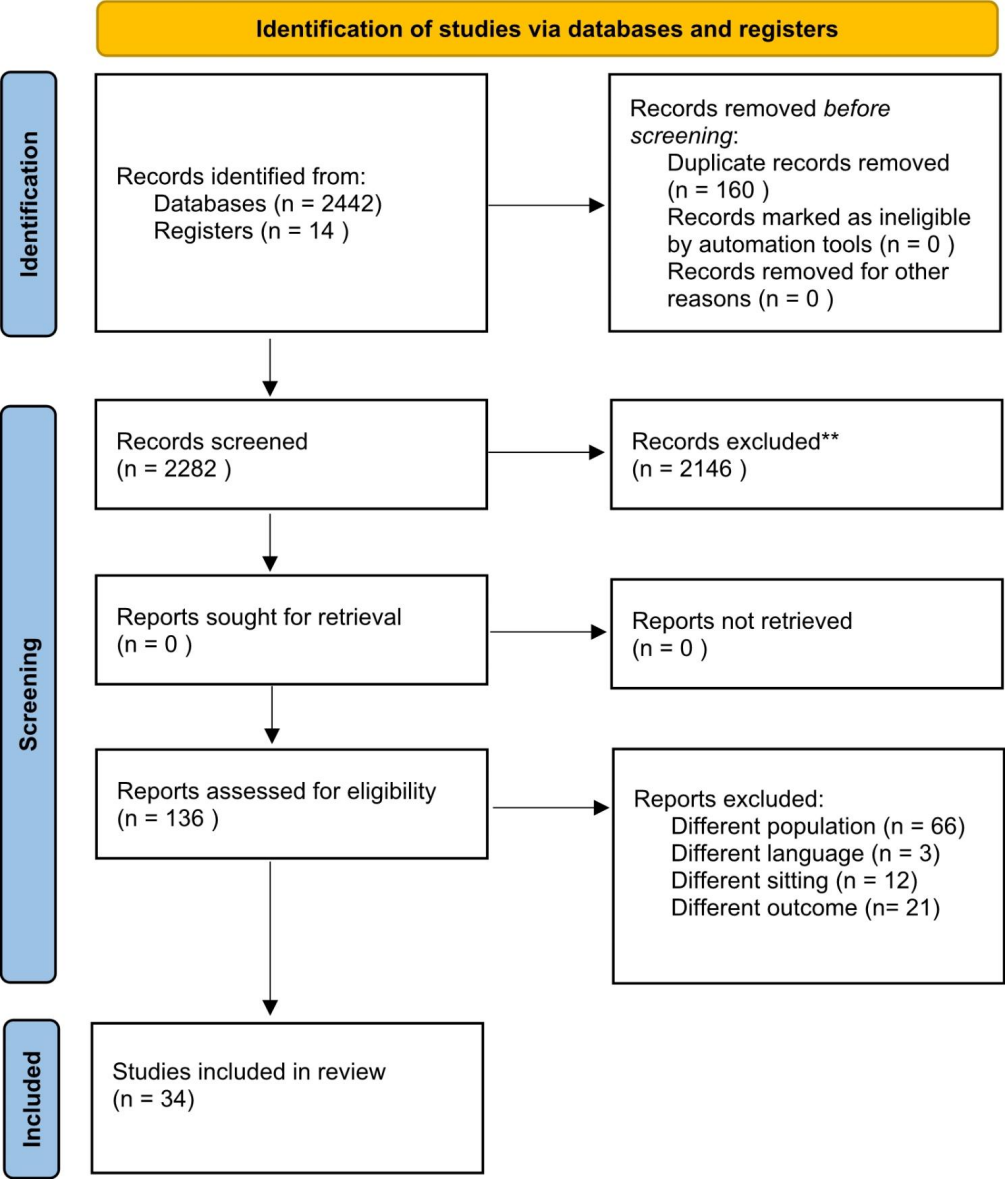
Flow chart of included and excluded studies

The researchers identified 2,442 articles through the systematic literature search. After removal of duplicates and title and abstract screening, 2,146 articles were excluded and the number remaining was 136. After the full-text screening, a total of 34 articles remained and were included in the final review. The PRISMA 2020 flow diagram was used as a template for reporting study inclusion (Fig. 1).

### Location

Seven of the 34 studies were conducted in **Nigeria** [12]; [13]; [14]; [15]; [16]; [17]; [18], four in **Ethiopia** [19]; [20]; [21]; [22], three in **Pakistan** ([23]; [24]; [25]), four in **Saudi Arabia KSA (**[26]; [27]; [28]; [29], two in **India** ([30]; [31]), two in the **USA** ([32]; [33]) and one each in the **UK** [34], **Ghana** [35], **Magnolia** [36], **Yemen** [37], **Tanzania** [38], **Afghanistan** [39], **Lebanon** [40], **China** [41], **Cameron** [42], **Canada** [43], **Kenya** [44] and **Russia** [45].

### Study design

Thirty-one of the articles reported studies of a cross-sectional design (quantitative studies) [12–18]; [20–35]; [37–44]; three were qualitative studies [36]; [19]; [45].

More detailed characteristics of the included studies are presented in Table 3 (supplementary material 1).

### Assessment of quality

Two authors (HA and IM) contributed independently to appraisal, and any disagreements were solved by discussion. Scores of either 0 or 1 point were given per criterion. One point was given if the answer was YES (the item was mentioned in the study) and zero if the answer was NO or UNCLEAR (the item was not mentioned or was unclear). All studies (low and high quality) were included in the review, and the study quality would be used to inform the results and the conclusions made throughout. The quality assessment results are shown in (Table 4) and (Table 5).

**Table 4:** Quality assessment results (Cross-sectional studies)

Study	Inclusion criteria	Subjects and Settings	Exposure measure	Measurement of the condition	Confounding factors	Dealing with confounding factors	Outcomes measure	Statistical analysis	Total quality scores
[12]	Yes	Yes	Yes	Not applicable	No	Unclear	Yes	Yes	6/8
[13]	Yes	Yes	Yes	Not applicable	No	Yes	Yes	Yes	7/8
[14]	Yes	Yes	Yes	Not applicable	No	Unclear	Yes	Yes	6/8
[15]	Yes	Yes	Unclear	Not applicable	No	Unclear	No	Yes	4/8
[16]	Yes	Yes	Yes	Not applicable	No	Unclear	Yes	Yes	6/8
[17]	Yes	Yes	Yes	Not applicable	No	Yes	Yes	Yes	7/8
[18]	Yes	Yes	Yes	Not applicable	No	No	Yes	Yes	6/8
[20]	Yes	Yes	Yes	Not applicable	No	Yes	Yes	Yes	7/8
[21]	Yes	Yes	Yes	Not applicable	Unclear	Yes	Yes	Yes	7/8
[22]	Yes	Yes	Yes	Not applicable	Yes	Yes	Yes	Yes	8/8
[23]	Unclear	Yes	Unclear	Not applicable	No	No	Yes	Unclear	3/8
[24]	No	Yes	Unclear	Not applicable	No	Yes	Yes	Yes	5/8
[25]	No	Yes	Unclear	Not applicable	No	No	Yes	Yes	4/8
[26]	Yes	Yes	Unclear	Not applicable	No	Unclear	Yes	Yes	5/8
[27]	Yes	Yes	Yes	Not applicable	No	No	Yes	Yes	6/8
[28]	No	Yes	Unclear	Not applicable	No	No	Yes	Yes	4/8
[29]	Yes	Yes	Yes	Not applicable	Unclear	Yes	Yes	Yes	7/8
[30]	Yes	Yes	Yes	Not applicable	No	Unclear	Yes	Yes	6/8
[31]	Yes	Yes	Yes	Not applicable	No	No	Yes	Yes	6/8
[32]	Yes	Yes	Yes	Not applicable	No	Unclear	Unclear	Yes	5/8
[33]	Yes	Yes	Unclear	Not applicable	No	Unclear	Yes	Yes	5/8
[34]	Yes	Yes	Yes	Not applicable	No	Unclear	Yes	Yes	6/8
[35]	Yes	Yes	Yes	Not applicable	No	No	Yes	Yes	6/8
[37]	Yes	Yes	Yes	Not applicable	Unclear	Yes	Yes	Yes	7/8
[38]	Yes	Yes	Yes	Not applicable	No	Unclear	No	Yes	5/8
[39]	Yes	Yes	Yes	Not applicable	No	Yes	Yes	Yes	7/8
[40]	Yes	Yes	Yes	Not applicable	No	No	Yes	Yes	6/8
[41]	Yes	Yes	Yes	Yes	No	Unclear	Yes	Yes	6/8
[42]	Yes	Yes	Yes	Not applicable	No	No	Yes	Yes	6/8
[43]	Yes	Yes	Yes	Not applicable	No	Unclear	Yes	Yes	6/8
[44]	Unclear	Yes	Yes	Not applicable	No	Unclear	Yes	Yes	5/8

**Table 5:** Quality assessment results (Qualitative studies)

Study	Philoso-perspective and method	Method and research question or objectives	Method and data collection methods	Method and data Representation and analysis	Method and results interpretation	Locating the researcher culturally or theoretically	Influence of the researcher on the research, and vice- versa	Representation of participants and their voices	Ethical approval by an appropriate body	Relationship of conclusions to analysis, or interpretation of the data	Overall score
[19]	Yes	Yes	Yes	Yes	Yes	Unclear	No	Yes	Yes	Yes	8/10
[36]	Yes	Yes	Yes	Yes	Yes	Unclear	Yes	Yes	Yes	Yes	9/10
[45]	Yes	Yes	Yes	Yes	Yes	Unclear	Yes	Yes	Yes	Yes	9/10

Thirty papers [12–14]; [16–22]; [24]; [26, 27]; [29–45] were considered to be of high quality. The remaining four were considered to be of low quality, mainly owing to lower representativeness of inclusion/exclusion criteria of study participants, outcome measures and statistical analysis.

### Knowledge, attitude and practice of infection control guidelines

For the purposes of this review, KAP among the study participants refers to the level of compliance related to the implementation of IPC guidelines among laboratory staff and includes one of the following definitions [50, 51]:

#### Knowledge

Information possessed on the IPC guidelines.

#### Attitudes

Opinion on and behaviour towards the IPC guidelines.

#### Practices

Observable actions towards the IPC guidelines.

Of the 34 included studies, the KAP of IPC and biosafety guidelines were identified and grouped into several themes.

#### (1) Knowledge of IPC precaution

There were no standardised criteria for classifying knowledge as poor, moderate or good across studies. However, it has been observed that the term ‘poor knowledge’ was generally used when < 50% of participants had adequate knowledge on the information about the IPC guidelines. Similarly, the terms ‘moderate’ and ‘good’ knowledge were used when the participants with adequate information about the guidelines were between 50 and 70% and > 70%, respectively, and this was also applied for the remaining themes as follows.

Knowledge was examined in 17 studies. Ten studies [18]; [22]; [20]; [21]; [29]; [26]; [28]; [31]; [40]; [38] reported good knowledge of IPC precautions among laboratory staff. Ndu et al. [18] attempted to differentiate between the knowledge among two groups of healthcare professionals: doctors and laboratory staff. Although the authors found there were differences between the two groups on the knowledge of components of IPC, both showed a good level of knowledge (76.2% in doctors and 67.6% in laboratory staff). About 55.4–84.7% of laboratory staff had a good level of knowledge as reported in studies [22]; [20]; [21] and it should be clarified that the number of laboratory staff included in these studies was very low compared to other healthcare workers (13/150; 29/49; 58/605), respectively. The reported results of knowledge in studies [29]; [26]; [28]; [31]; [38] were (84%; 66%; 81.97%; 75%; 82%), respectively. Because a small number of laboratory staff (10) participated in study [26], it may not be a good representative of laboratory staff. Rabaan et al. [28] assessed the knowledge of IPC policies and guidelines, but it is considered to be a study of low quality because it has no information regarding the inclusion/exclusion criteria of its study sample. Almost all the technicians were knowledgeable about the IPC precautions (100%) in the Lebanese study [40].

In contrast, four studies [15]; [13]; [12]; [25] reported moderate knowledge of IPC precautions among laboratory personnel. In Fadeyi et al.’s study [15], only 58.2% of the participants were aware of safety precaution principles, while in Ibeziako and Ibekwe’s study [13] about 50.4% of the respondents were aware of IPC precautions. The results of Izegbu et al.’s study [12] showed that only 20.8% of the participants had heard of the IPC precautions and only 37.5% of these could define and state their objectives. Only 51% of participants knew that the standard method of discarding needles is without recapping [25]. However, this study has flaws in its quality assessment tool because no information regarding the inclusion/exclusion criteria of its study sample was reported. Furthermore, the instrument used for data collection was not pretested to check its validity and reliability.

The remining three studies [30]; [37]; [35] reported poor level of knowledge among laboratory staff. The reported results of knowledge in study [30] was (32%), in Akagbo et al. the reported level was (37.0%) [35], and only 38% of respondents had a good level of knowledge in the Yamani study [37]. It is important to highlight that the findings of study [35] were drawn from only five laboratory members of staff out of 100 healthcare workers.

The participants of one qualitative study included in this review claimed that many infection control decisions are made by those who have a non-medical background or are non-knowledgeable in infection control. In addition, all the study participants acknowledged their poor knowledge of infection control and reported that IPC is not well taught at the under- and postgraduate levels of education. Poor knowledge on disinfection and sterilisation were also reported because the standards and guidelines for disinfection and sterilisation have not been updated in the laboratory [36].

Another qualitative study showed that laboratory staff were most knowledgeable about tuberculosis IPC guidelines because they believed wearing hospital-laundered lab coats and disposable shoe coverings was protective against TB transmission. Participants also described the necessity of showering and changing clothes so they did not carry the bacillus home [45.

##### Immunisation against infectious diseases

The assessment of the immunisation status of laboratory staff has been reported in eight studies.

In KSA, 60% of respondents who worked in laboratories had been vaccinated against hepatitis B [26], and 87% had received a smallpox vaccination in their lifetime [33].

However, in Nigeria, the situation is different. The findings revealed that the awareness of HBV vaccine is not good enough, in that only 46.2% were aware of the availability of the HBV vaccination in their workplace even though 72.3% of participants were willing to be vaccinated [15]. It was further found that 91.5% of participants were not immunised against HBV [12].

In India, the results were similar, in that 91.5% were not immunised against HBV [30]. Meanwhile, in Pakistan, 90.9% of participants were vaccinated against HBV [25].

A Kenyan study’s authors found that all the staff participating in the study were aware of the importance of the vaccination, but because it was optional in their institution, they chose to remain unvaccinated [44]. Meanwhile in Afghanistan, 78.0% of participants were vaccinated despite the fact that vaccination against HBV is not covered by the government and healthcare workers have to pay from their own funds to receive this vaccination [39].

##### Training on IPC guidelines

Twelve studies’ authors reported the results of training on IPC precautions.

In Nigeria, only 13.8% had received training on universal precautions [13], and the authors investigated how low and unequal levels of training among staff contribute to the poor knowledge of and compliance with the precautions. The training level was similar between medical doctors and laboratory staff (53.1% of medical doctors and 58.1% of laboratory staff). However, in Ndu et al.’s study [18], 73.5% of the laboratory staff received training on wearing and removing PPE, which may contribute to the low use of PPEs among doctors compared to laboratory staff.

In the study of Desta et al. [22], participants who had undertaken IPC training amounted to 35.33%, and there was an association between training and practice. Only 36.8% of the participants had taken biomedical waste management training, which led to the overall unsatisfactory level of KAP scores in the study [20].

Training status was reported in two Saudi studies. For instance, 68% of participants reported receiving training in laboratory safety either through a course during college education or through training workshops in their workplace [29]. However, the results showed that some of the unacceptable behaviours in laboratories were associated with lack of training in IPC precautions. Of the participants, 23.06% reported having received no training [28], and when the participants were asked to identify factors that contribute to the spread of infection in the hospital, 51.73% reported no infection control training program as a factor.

A Tanzanian study revealed that the percentage of the study sample who received training on universal precautions was 98.5%, and the previous training was significantly associated with good practice (P < 0.001) [38].

These findings match the results reported in Pakistan, where no formal biosafety training had been provided to 84.2% of the participants [24]. In Ghana it was reported that only 48% of participants had regular training in IPC precautions [35], and in Yemen 67% and 32% of private and public laboratory staff had received training, respectively [37]. No associations between training and practice were reported in all three studies [24]; [35]; [37].

#### (2) Attitude of IPC precautions

The attitude of laboratory staff towards IPC were examined in nine studies.

Good level of attitude was reported in four studies [31]; [40]; [27]; [29]. The good attitude level was observed in three departments in the laboratory: 83.3% in the pathology department, 75% in the biochemistry department and 100% in the microbiology department [31]. Only 8 of the 73 (11.0%) technicians showed some behavioural lack inside the laboratory: eating, drinking, smoking or pipetting with their mouths [40]. In Khan et al.’s study [27], although the majority of respondents demonstrated good behaviours towards the use of IPC protective measures (58.8%), they displayed poor behaviours towards their active participation in infection control programs (24.2%). Meanwhile, in Khabour et al.’s study [29], only 24.2% of participants were willing to eat, drink or use gum, 18.3% used cosmetics and 24.6% used their mobile phones in the laboratory.

Three studies reported moderate level of attitude [15]; [20]; [21]. In Fadeyi et al.’s study [15], 60.0% of participants were willing to eat and drink in the laboratory and the reported attitude level in study [20] and [21] were (66.2%; 66.1%), respectively.

Poor attitude level can be observed in two studies [21]; [31]. In Izegbu et al.’s study [12], 45.6% of the participants ate in the laboratory and 47.0% of them stored food and water in the refrigerators meant for the storage of body fluids and chemicals, attitudes that indicate a disregard towards IPC and safety precautions. The results of Zaveri et al.’s study [30] surprisingly matched exactly the findings from Izegbu et al.’s study [12].

##### Perception of risk

Only three studies in this review related to risk perception among laboratory staff.

Only 23% of laboratory workers in the UK thought they were at some risk of HIV infection in their occupational setting; this low percentage may relate to the high knowledge of safe working practice and practical working experience, or they worked in a safe lab using safe practices [34]. A study assessed prion disease risk perception among laboratory staff found that 18% believed that they were at risk of prion transmission when processing prion-associated specimens and 81% would be more comfortable processing specimens if safety guidelines existed and were used in their laboratory [43]. One qualitative study concerned healthcare workers’ perceptions on occupational risk of HIV transmission [19]. Alemie [19] reported that all the participants were aware of the risk of acquiring HIV in healthcare settings and all of them were worried about the inadequacy of protective materials required to prevent HIV transmission, which was mentioned as the main reason for perceived high risk.

#### (3) Practice of IPC precautions

The majority of studies (23) in this review were on laboratory staff practice of IPC precautions.

Six studies were Nigerian, and the authors of those included in this review assessed how IPC precautions were practised in laboratories. Poor practice results were reported in two studies [12] (43%) and [17] (45.6%). Moderate findings were reported by Fadeyi et al. [15] in that about 69.2% of participants wore gloves when handling samples, and in Sadoh et al.’s study [14], 63.8% of participants always used PPE. The findings in Ndu et al.’s study [18] demonstrated that laboratory staff reported good practice and greater use of PPE such as gloves and coveralls than doctors (100% and 35%, respectively). The same good practice level was reported in Ibeziako and Ibekwe’s study [13] in that gloves were used by 86.6% of respondents, while only 43.9% of them practised appropriate hand washing.

One Ethiopian study showed a good level of practice [20] (77.4%). However, two other studies [22] and [21] showed moderate results (57.3%) and (66.1%).

In KSA, it was revealed that only 27% of participants were using gloves all the time, while 48 (69%) were doing so only occasionally [26]. It was further documented that 10–25% of injuries in the laboratory occurred while recapping a used needle [26]. Nevertheless, Khabour et al.’s study [29] demonstrated a good practice level among laboratory staff, and the majority (> 80%) of participants followed guidelines for disposal of medical waste, decontamination of sample spills and use of protective lab coats and gloves, among other measures.

Indian studies reflected good practice levels [30] and [31]. All participants wore gloves during laboratory work [30], and 66.7%, 81.5% and 100% of participants in the pathology, biochemistry and microbiology departments, respectively, gave correct answers to the practice questions in the study questionnaire [31].

All three studies conducted in Pakistan demonstrated a poor level of practice. There was a lack of awareness of good laboratory practices reported in Nasim et al.’s studies [23] (because 46.2% of the participants did not use any kind of PPE, and almost 39.5% recapped used syringes regularly) and the practice level was 33.6% in [24]. Qazi et al.’s study [25] yielded poor results because 80.3% of 208 participants were recapping needles, which meant that 31.3% had experienced a needlestick injury while recapping.

The studies conducted in Lebanon [40], Kenya [44] and Tanzania [38] showed good levels of application. In them, 93.2% of participants wore gloves while working in the laboratory [40], 97.8% used PPE, gloves, overalls, gumboots, mouth masks and other protective equipment when handling medical waste [44] and 77.0% applied universal precautions [38].

Conversely, the studies from Yemen [37] and Ghana [35] revealed a poor application level and the study from Afghanistan [39] revealed moderate level. In Afghanistan, 57.8% of respondents reported that they always recapped the needle after giving an injection [39], while in Yemen, only 32% of respondents had good practice of IPC precautions [37]. Only 50% of respondents always protected themselves from injections, and about a quarter of the respondents did not recap needles after use as reported in Ghana [35].

The participants of Ider et al.’s study [36] conducted in Mongolia perceived that hand-hygiene practice among health professionals of Mongolia was low. They also wondered why, despite most hospitals conducting staff hand-hygiene training once or twice a year, hand-hygiene practice remained poor. The main reasons for this may be the unavailability of hot water and sinks and a poor supply of soap, poor supply of alcohol-based hand sanitisers and skin care products, and high workload of health professionals [36].

In one study conducted in China, the authors aimed to assess the infection control practices among COVID-19-infected healthcare workers [41]. Before the COVID-19 outbreak, 53.4% of respondents always followed the procedure for wearing and removing PPE, 66.0% always wore masks and 51.5% wore gloves in their routine work. However, approximately 41.8% of participants thought their infection was related to protective equipment and utilisation of common equipment (masks and gloves), either owing to inadequate provision of PPE or to insufficient protection provided by the PPE they had.

Poor application of tuberculosis IPC guidelines was reported in Woith et al.’s study [45] in Russia. Poor application concerned the use of respirators and masks because they are uncomfortable especially during hot weather, wearing respirators interfered with using microscopes in the lab and the quality of the respirators available at their facilities was poor.

##### Exposure and post-exposure prophylaxis

Ten articles’ authors reported exposure to injuries and post-exposure prophylaxis (PEP) following injuries.

In Nigeria, 53.23% of the participants had had cuts or punctures from needles and were treated in the laboratories [12]. Although 94% of the laboratories had first aid boxes, only 28.78% of the staff made use of these [12]. In Fadeyi et al.’s study [15], despite the fact that 79.2% of respondents were aware of the availability of PEP for HIV and HBV, only 1.5% positively responded to presenting themselves and received PEP following any laboratory accidents [15]. Half of the laboratory workers who participated in the study [16] had experienced needle pricks, and only 25.7% of exposures were reported to the staff clinic.

Four of the seven participants in Alemie’s study [19] in Ethiopia had experienced accidents: needlestick injuries or exposure to blood or other body fluids, and their explanations of the incidents indicated the accidents were frequent. Many of the injuries/accidents were followed by commencement of PEP, which, however, was mentioned by some to be less practised although they were well aware of it [19].

No percentages of accidents were reported according to studies [23] and [24], but 83.4% and 89.3% of laboratories did not maintain any accident records, respectively. In Rabaan et al.’s study [28], about 31.3% of participants had experienced a needlestick injury while recapping; however, only 24.2% of participants who experienced an injury were aware that they should take PEP.

A similar situation was noted in a Saudi study, where 74% of participants had a history of needlestick injuries, and only 21% of the 74% reported the injuries to the hospital authority [26].

In India, 53.23% of the participants had been injured by needles and sharp instruments. However, only 28.78% of them made use of first aid supplies after their injury [30].

A Cameroonian study’s authors [42] reported exposure and PEP and agreed with the findings of the Indian study [30]. This showed that a high proportion of participants (58%) had poor knowledge of PEP and 60.6% had a positive attitude towards PEP. About 50.9% of all participants had had at least one occupational exposure, but only 19.1% of PEP incidents were recorded among exposed participants.

The reported data on occupational accidents/injuries rely on the participants’ memories of past exposure, which may therefore be prone to recall bias.

#### (4) Associations among knowledge, attitude and practice

Only the authors of four of the included studies examined the associations/correlations among KAP. Three studies found a significant correlation between knowledge and practice regarding IPC precautions (r = 0.76, p < 0.001) [38]; [23]; [22]. The correlation between knowledge and attitude was significant (r: 0.12; P < 0.001) [27], and there was an association between adherence to IPC guidelines and the practice of infection prevention [22].

#### (5) Barriers and facilitators to implementation

One quantitative and one qualitative study authors explored barriers and facilitators to the implementation of IPC guidelines.

It has been found that the factors that positively promote consistent adherence were: education in standard precautions, providing facilities with PPE and strong management support for safety. An increase in workplace demands and expectations negatively affected consistent adherence [32]. In Mongolia, a qualitative study’s authors assessed the perceptions of laboratory staff regarding the main barriers and challenges to implementation of effective infection control in the hospital. They found that poor IPC education, limited laboratory capacity, poor disinfection and sterilisation, and low compliance with hand hygiene were the major barriers to implementation [36] (see Table 4). Although the researchers examined issues from the participants’ perception, there were shortcomings in how this study was conducted, and it could have been improved using large-scale quantitative and mixed-method investigations.

## Discussion

This review concerned the level of knowledge of, attitudes to and practice of IPC precautions/guidelines among staff working in laboratories in different countries.

This has been done through unpacking the KAP in particular themes, and the definition of each theme was identified from the studies included in this review. Several differences of KAP were observed between and within countries. Generally, the available evidence shows that there was good^1^ knowledge, good^1^ attitudes and moderate^2^ immunisation status, but there was still poor^3^ practice of IPC precautions among laboratory workers. Evidence is lacking on risk perception, and it was low based on the available articles. Exposure to blood and body fluids through cuts or punctures from needles and sharp instruments was high among laboratory staff; despite the high incident rate, the reporting of these accidents to the management team and use of PEP was low. There was an inadequate level of training received among laboratory staff, and some studies revealed a strong association between training and knowledge and the thorough practice of IPC. Although the evidence was not abundant, there is a clear association among KAP. The lack of guidelines, the poor access to safety equipment (PPE), the lack of training and education and the immense pressure of emergency situations were the main barriers highlighted in this review. The findings show that there is a need to improve the availability of guidelines, the availability of PPE and the provision of regular training on IPC guidelines.

Different definitions of knowledge were used in different studies, which reflects the lack of stable policies and guidelines, and this may be because different IPC recommendations are made by the Centres for Disease Control and Prevention and the World Health Organisation (WHO). Similarly, different levels of knowledge were reported in the included studies. However, some of them were considered to be of low quality according to the JBI-CAT, and in addition the number of laboratory staff included in the study was very low.

The findings on the laboratory staff’s attitudes towards the IPC guidelines were more focused on eating, drinking, storage of food in refrigerators and the use of mobile phones. None of the researchers reported the reasons behind this poor behaviour. There is a need to clarify the reasons behind these risky behaviours and poor attitudes, because they must be urgently addressed to prevent the establishment of a poor work culture.

In line with the reason of poor practice reported earlier, it should be clarified that most of the studies whose authors assessed practice in this review were limited by a self-reporting method. This method may have produced a less favourable picture of practice than is actually the case, and the participants may tend to overestimate the extent to which they practice and comply with IPC precautions.

Evidence on risk perception was very low in this review, very few laboratory staff members were included in the data and it is difficult to draw meaningful conclusions from such sparse data. The same applies to the associations among KAP. Although there is a clear association reported in this review, the available evidence was greatly lacking and so more studies on this area are recommended.

The lack of reporting on the incident rate and on the use of PEP may be owing to the lack of awareness of the importance of PEP, fears of stigmatisation and job insecurity [14]. According to the WHO, PEP can reduce the risk of HIV infection by over 80% if started soon after exposure [48]. Therefore, hospital authorities should establish a continuing health education programme to inform laboratory staff on IPC measures with particular attention on the immediate action to take after injuries, reporting injuries and the use of PEP. In addition, setting up a monitoring team is needed to actively keep looking at all occupational injuries and exposures, so as to guarantee that they are managed and reported properly.

The overall training level was unsatisfactory, and it has been shown from the aforementioned evidence that training programs for laboratory staff can affect their adherence to, knowledge of, behavior towards and practice of IPC precautions. It is therefore recommended that they receive enough training regarding IPC precautions and examination before gaining the license to practice a laboratory profession.

Similar to this review, a recent review about the occupational hazards among healthcare workers in Africa showed a lack of PPE as a common reason for poor practice [47]. This indicated that there is a need for national policies to address low availability and in some cases the complete absence of PPE in many low-income countries. The findings of the Ghanaian study [39] highlighted how complying with the IPC precautions sometimes interferes with workers’ ability to provide care. The study reflected how the warmer climate in such countries meant that healthcare workers were exposed to heat stress, which may limit their compliance and may also make the use of PPE more uncomfortable than in cooler climates and could even be life-threatening [46]. Consequently, the standards for the production of PPE should take warmer climates in these countries into consideration to promote adherence.

There is a need for more mixed-methods studies to assess the KAP of laboratory staff to reduce biases during the data collection. Furthermore, the majority of articles in this review were focused on either standard or universal precautions with very few mentions of both of them together. Thus, studies on both kinds of precautions are required because they are equally important and recommended by the WHO. Larger-scale studies are needed to collect more evidence about risk perception among laboratory staff.

The study had some limitations. Some of the included studies in this review were focused on laboratory staff alone as participants, while others were focused on all healthcare workers such as nurses and doctors as well as laboratory staff; therefore, certainly a higher level of KAP will have been attained and reported in the ones that were only focused on laboratory staff than in other broader studies. In addition, because a narrative synthesis approach was followed and not enough numerical data were available in this review, there was no assessment of publication bias carried out because it does not allow funnel plots to be presented. Finally, only studies published in the English language were included. Thus, the potential language bias is considered to be a limitation of this review.

## Conclusion

This systematic review advanced the current knowledge regarding the level of KAP regarding IPC precautions among laboratory staff. It clearly shows via evidence that there is a gap among KAP, which indicates that laboratory staff are at high risk of acquiring infections in the workplace. These findings suggest that training (including IPC precautions, safety policies, safety equipment and materials, safety activities, initial biohazard handling, ongoing monitoring and potential exposure) of laboratory staff to increase their knowledge about IPC precautions could improve their use of these precautions. It is also recommended that the administration or policy makers in the hospital should provide a suitable environment for the implementation of the IPC guidelines.

## Electronic supplementary material

Below is the link to the electronic supplementary material.

Supplementary Material 1

**Additional file 1**: JBI Critical Appraisal Checklist for Cross-sectional Studies

**Additional file 2**: JBI Critical Appraisal Checklist for Qualitative Research

## Data Availability

The datasets used and/or analysed during the current study are available from the corresponding author on reasonable request.

## Footnotes

1 The majority of studies reported results > 70%.

2 The reported results vary between > 50% and < 70%.

3 The majority of studies reported results < 70%.

## List of Abbreviations

IPC: Infection prevention and control
KAP: Knowledge, attitude and practice
PPE: Personal protective equipment
KSA: Kingdom of Saudi Arabia
UK: United Kingdom
CDC: Disease Control and Prevention
WHO: World Health Organisation
