# Supplementary material for: Knowledge, attitude and practice of infection prevention and control precautions among laboratory staff: a mixed-methods systematic review
Source: Antimicrob Resist Infect Control. 2023 Jun 13;12:57. doi: 10.1186/s13756-023-01257-5 (PMC10262112; doi:10.1186/s13756-023-01257-5)
Supplement: Supplementary file 1 — Supplementary Material 1 [file 13756_2023_1257_MOESM1_ESM.pdf]

Table 3: Data extraction form

Author/ Year	Main focus	Method	Country	Sample	Outcome measures	Results
[12] Izegbu et al., 2006	Attitudes, perception and practice of workers in laboratories in the two Colleges of Medicine and their teaching hospitals as regards universal precaution measures	Cross-sectional study	Nigeria	154 participants Medical laboratory scientist.	Eating in the laboratory, storage of food and water in the refrigera-tor meant for body fluids, drugs chemicals or other specimens, application of cosmetics, smoking or sniffing, cutting of fingernails with teeth or putting the biro in the mouth, wearing of hand gloves, puttingon of laboratory coats, immunization against hepati-tis B virus, washing of hands after removal of hand gloves and wearing of gloves.	All the participants wear gloves during laboratory work. 20.8% of the participants had heard of it. 45.6% of the participants eat in the laboratory, 47.0% of them store foods and water in the refrigerators meant for storage of body fluids and chemicals, 31.5% of them put on cosmetics in the laboratory, 91.5% are not immunized against hepatitis B virus (HBV). 82.0% of the participants do not feel that the use of masks is necessary in laboratory 53.23% (n=82) of the participants had had cuts or punc-tures from needles, surgical blades, sharp device.
[13] Ibeziako and Ibekwe, 2007	Knowledge and practice of universal precaution	Cross-sectional study	Nigeria	246 participants (34 lab staff)	Knowledge and practice of infection control policy.	124 (50.4%) of the respondents were aware of universal precaution, while 88 (35.8%) knew the correct definition of universal precaution. 34 (13.8%) had received training on universal precaution. Hands gloves were used by 86.6% of the respondents and 43.9% practiced appropriate hand washing. Training significantly associated with knowledge (P=0.006).
[14] Sadoh et al., 2006	Practice of Universal Precautions among Healthcare Workers	Cross-sectional study	Nigeria	433 participants (93 lab staff)	Practice of recapping and disposal of used needles, use of barrier equipment and handwashing.	About a third of all respondents always recapped used needles. Compliance with nonrecapping of used needles was highest among trained nurses and worst with doctors. 63.8% always used PPE. A high percentage (94.6%) of participant observed handwashing after handling patients.

[15] Fadeyi et al, 2011	Awareness and practice of safety precautions among healthcare workers in the laboratories	Cross-sectional study	Nigeria	130 participants	Awareness of safety precautions and availability of protective equipment in the laboratory. Practice and attitude related to safe laboratory practice such as use of protective equipment, handling of contaminated items and post-laboratory accidents/injury measures.	58.5% of the respondents were aware of Safety Precaution. Participants attest to availability of various safety devices and equipment including hand gloves (86.2%), disinfectants (84.6%), HBV immunisation (46.2%) and post exposure prophylaxis (PEP) for HIV and HBV (79.6%). Attitude to safety is unsatisfactory as 60.0% eat and drink in the laboratory, 50.8% recap needles and 56.9% use sharps box. Even though 83.1% are willing to take PEP, only 1.5% will present self following laboratory injury.
[16] Isara and Ofili, 2012	Prevalence of Occupational Accidents/Injuries among Health Care Workers	Descriptive cross-sectional study	Nigeria	167participants (20 laboratory workers)	Socio-demographic data of the HCWs and their exposures to needle pricks and other occupational accidents.	. 10 (50.0%) laboratory workers had had needle pricks, Only 43 (25.7%) of respondents reported to the staff clinic after sustaining accidents/injuries.
[17] Bello et al., 2016	Health Workers' Knowledge, Attitude and Practice towards Hepatitis B Infection	Cross-sectional study	Nigeria	108 participants (13 laboratory technicians)	The relationship between knowledge, attitude and practice among health workers towards hepatitis B infection.	There is a gap in knowledge and lack of compliance to infection control and preventive measures among health-care professionals
[18] Ndu and Arinze-Onyia, 2017	Standard precaution knowledge and adherence: Do Doctors differ from Medical Laboratory Scientists	Ccross sectional study	Nigeria	143 doctors and 136 medical laboratory staff	Demographical variables, knowledge and adherence to SP and associated factors.	General knowledge of SP was high,76.2% in doctors and 67.6% in MLSs. Use of personal protective equipment as well as safe handling of contaminated equipment or surfaces was higher amongst doctors. Even though more than half of respondents in both groups, 53.1 % among doctors and 58.1% among MLSs had received training on standard precautions, this did not reflect in the practice. MLS reported more use PPE (100% in MLS and 35% of doctors). Recapping of syringes was higher amongst doctors (63.6%) than MLS (55.1%). Constraints that affected SP included non-availability of PPEs and emergency situations for both groups.

[19] Alemie, 2012	Exploration of healthcare workers' perceptions on occupational risk of HIV transmission	Qualitative study	Ethiopia	7 participants (1 laboratory staff)	The risks related to their work, their experience of HIV related hazards and their general views on the transmission of HIV.	All the respondents were aware of the risk of acquiring HIV in healthcare settings. Some had experienced accidents that made them take post-exposure prophylaxis..They also expressed their feelings that their workplace was not the best place to work at.
[20] Deress et al., 2018	Assessment of Knowledge, Attitude, and Practice about Biomedical Waste Management and Associated Factors	Cross-sectional study	Ethiopia	296 participants (49 laboratory staff)	Sociodemographic and HCF related factors, knowledge, attitude, and practice.	56.8%, 66.2%, and 77.4% had adequate knowledge, favorable attitude, and adequate practice score, respectively. Less than one-third (30.7%) of the study participants were vaccinated for hepatitis B virus. Regarding previous training, only 109 (36.8%) had taken BMWM training.
[21] Sahiledengle et al., 2018	Infection Prevention Practices and Associated Factors among Healthcare Workers	Cross-sectional study	Ethiopia	605 participants (58 laboratory staff)	Awareness on infection prevention (IP) components Presence of hand washing facility. Availability of PPE. Ever had needle stick or sharp injury. Awareness on availability PEP available daily/weekly. Knowledge of HCWs on infection prevention measures. Attitude of HCWs toward infection prevention practices.	66.1% health care workers had good infection prevention practices. Having good knowledge on infection prevention meausures (AOR =1.53), having positive attitude towards infection prevention practices (AOR=2.03).
[22] Desta et al., 2018	Knowledge, practice and associated factors of infection prevention among healthcare workers	Cross-sectional study	Ethiopia	150 participants (13 laboratory technicians)	The dependent variables studied were knowledge and practice of healthcare workers towards infection prevention. Whereas, the independent variables include institutional factors (training about infection prevention, availability of infection prevention supplies).	84.7% of healthcare workers were found to be knowledgeable but only 86 (57.3%) of respondents demonstrated a good practice on infection prevention. Healthcare professionals who have taken Infection prevention training were 35.33%. In-service training, availability of infection prevention supplies and adherence to infection prevention guidelines was also associated with the practice of infection prevention.
[23] Nasim et al., 2010	Practices and Awareness regarding Biosafety Measures among Laboratory Technicians Working in	Cross-sectional study	Pakistan	253 laboratory technicians	Awareness and biosafety measures taken by hospital-based laboratory technicians during their routine work in clinical laboratories such as unsafe work practices	46.2% of the laboratory technicians did not use any kind of PPE, and almost 39.5% of the respondents recapped used syringes regularly. Although mouth pipetting is considered obsolete, 38% of the technicians continue to do so for various

	Clinical Laboratories				(e.g., eating or drinking in laboratories), mouth pipetting of biological samples, use of PPE, and proper disinfection, specimen handling, collection, and processing.	purposes. Additionally, accident records were not maintained in 83.4%. No formal biosafety training had been provided to 85% of the respondents.
[24] Nasim et al., 2012	Biosafety perspective of clinical laboratory workers	Cross-sectional study	Pakistan	1,782 laboratory technicians	The awareness of biosafety measures and the practices performed by laboratory technicians during their routine laboratory work.	28.4% of the laboratory technicians from Punjab, 35.7% from Sindh, 32% from Balochist an and 38.4% from Khyber Pakhtoon Khawa (KPK) did not use any PPE. Furthermore, 30.7% of the respondents said they discard used syringes directly into municipal dustbins. The majority (66.7%) claimed there are no separate bins for sharps, so they throw these in municipal dustbins.<br>Accident records were not maintained in 83.4%. No formal biosafety training had been provided to 84.2% of the respondents.
[25] Qazi et al., 2016	Comparison of awareness about precautions for needle stick injuries	Cross-sectional study	Pakistan	198 participants (58 laboratory technicians)	Level of awareness amongst health care workers<br>Adopting precautionary measures i.e. using gloves for standard procedures, know how of standard method of discarding needles i.e. without recapping, practicing method of discarding needles, awareness of Hep B spread through NSIs, awareness of Hep C spread through NSIs, awareness of HIV spread through NSIs, receiving booster dose and reason of not getting vaccinated.	51 % knew that the standard method of discarding needles is without recapping. 80.3 % were still recapping needles. 90.9 % HCWs were vaccinated against Hepatitis B.<br>The prevalence of NSIs was 50 % and out of these, 31.3 % had experienced an NSI while recapping. Only 24.2 % people who experienced an NSI were aware enough to take post exposure prophylaxis, a greater number of which were the lab technicians 11 (45.8 %)
[26] Alam, 2002	Knowledge, Attitude and Practices Among Health Care Workers on Needle-Stick Injuries	Cross-sectional study	Kingdom of Saudi Arabia	70 participants (10 laboratory staff)	Demographic data, job category, HBsAg, anti HCV and HIV status of the health care worker. The knowledge and use of preventive measures regarding needle-stick injuries.	74% had a history of needle -stick injuries and only 21% reported the injuries to the hospital authority. Only 66% were aware of Universal Precaution Guidelines. 60% had been vaccinated against hepatitis B, while 40% were not vaccinated against hepatitis B.

[27] Khan et al., 2014	Knowledge and attitude of healthcare workers about middle east respiratory syndrome	Cross sectional study	Kingdom of Saudi Arabia	153 participants (24 laboratory staff)	Demographic information of the respondents. The source of respondents' MERS knowledge. The knowledge of healthcare workers regarding MERS. The attitude of respondents towards MERS.	The correlation between knowledge and attitude was significant (correlation coefficient: 0.12; P <0.001. Although the majority of respondents showed positive attitude towards the use of protective measures (1.52 ± 0.84), their attitude was negative towards their active participation in infection control program (2.03 ± 0.97).
[28] Rabaan et al., 2017	Iinfection prevention and control in healthcare facilities in regards to Middle East Respiratory Syndrome	Cross sectional study	Kingdom of Saudi Arabia	607 Participants (233 laboratory staff)	Attitudes to, and awareness of, infection prevention and control policies and guidelines among healthcare workers.	Carelessness of healthcare workers was the top-cited factor contributing to causes of outbreaks (65.07% of total group), and hospital infrastructure and design was the top-cited factor contributing to spread of infection in the hospital (54.20%), followed closely by lack and shortage of staff (53.71%) and no infection control training program (51.73%). An electronic surveillance system was considered the most effective by staff (81.22%).
[29] Khabour, 2018	Assessment of biosafety measures in clinical laboratories	Cross-sectional study	Kingdom of Saudi Arabia	208 medical laboratory staff	Attitude, knowledge, and practices of medical laboratory staff.	About 89% of the sample had very good to excellent awareness about infection routes. The majority (> 80%) followed guidelines for disposing medical wastes, decontamination of sample spills, and use of protective lab coats, gloves, etc. However, among participants, 24.2% used to eat, drink or use gum, 18.3% used cosmetics and 24.6% used the mobile phone in the lab. About 18.4% reported that they continued working with a finger cut.
[30] Zaveri, 2012	Knowledge, attitude, and practice of universal work precautions amongst medical laboratory technicians	Cross-sectional study	India	154 laboratory technicians.	Attitude and practices of participants were included in the study. Participants were also scored on some items on biohazards and biosafety. Furthermore, participant's knowledge on the subject was sought by inquiring what they would do if they sustained injuries in the laboratory. The Hepatitis B vaccination	32% (20.8) of participants were aware of Universal Work Precaution. All the participants wear gloves during laboratory work but 81.2% wear a single pair. 17.5 % of the participants claimed to know what to do if exposed to infection. 45.6% of the participants eat in the laboratory, 47.0% of them store foods and water in the refrigerators, 31.5% of them put on cosmetics in the laboratory, 12.6% smoke in the laboratory, 10.0% cut their finger

					statuses were also determined.	nails with teeth in the laboratory. 91.5% are not immunized against hepatitis B virus (HBV). 99.0% of them do not take shower immediately after laboratory work. 82.0% of the participants do not feel that the use of masks is necessary in laboratory 53.23% of the participants had had injury.
[31] Wader et al., 2013	Knowledge, Attitude, Practice of Biosafety Precautions amongst Laboratory Technicians in a Teaching Hospital	Cross-sectional study	India	19 laboratory technicians	Safety Precaution, Disinfection of working area, Handling of blood and body fluid, Hand washing, Disposal of waste, Handling and transport of specimens, Dealing with sharp injury.	According to knowledge, in pathology 50% of study subjects were having average and 50% were having good scores while in biochemistry 25% had average and 75% had good scores and in microbiology 100% of study subjects had good grade. For attitude, in pathology dept 83.3% had average and 16.7% had good grades. In biochemistry 12.5% had poor grades, 75% had average grades and 12.5% had good grades. In microbiology 100% had good grades. For practice in pathology dept 16.7% had poor grades, 66.7% had average grades and 16.7% had good grades. In biochemistry 81.5% had average grade and 12.5% had good grades. In microbiology 100% of study subjects had good scores.
[32] Thomas et al., 2004	Factors Promoting Consistent Adherence to Safe Needle Precautions Among Hospital Workers	Cross-sectional study	The United State of America	1,454 participants (151Medical laboratory staff)	Consistent adherence, structural support, equipment availability , key leader support and HCW perceptions and attitudes.	Positive predictors of consistent adherence included infection control personnel hours per full-time–equivalent employee frequency of standard precautions education. facilities providing personal protective equipment and management support for safety (OR, 1.05). Negative predictor was increased job demands.
[33] Benzekri et al., 2010	Laboratory worker knowledge, attitudes and practices towards smallpox vaccine	Cross-sectional study	The United State of America	45 laboratory workers	Adherence to ACIP recommendations, assess potential barriers to vaccination and determine the influence of training on laboratory worker attitudes.	87% had received a smallpox vaccination in their lifetime; 73% received vaccination in the past 10 years. The main barrier to vaccination may be fear associated with possible vaccine adverse effects and a willingness to risk accidental infection rather than be vaccinated.

[34] Davidson and Gillies, 1993	Safe working practices and HIV infection: knowledge, attitudes, perception of risk, and policy in hospital	Cross-sectional study	The United Kingdom	1530 participants (170 laboratory staff)	Knowledge of safe working practices and hospital guidelines; attitudes towards patients with AIDS; perception of risk of occupational transmission of HIV; availability of guidelines.	All staff knew of the potential risk of infection from needlestick injury (98%, 904/922).<br>In all, 32% of staff (303/958) indicated that they thought they were at some risk of HIV infection in their occupational setting, only 23% of doctors and laboratory workers and 38% (48/127) of nurses considered themselves to be at risk.
[35] Akagbo, 2017	Knowledge of standard precautions and barriers to compliance among healthcare workers	Cross-sectional study	Ghana	100 participants (5 lab technicians)	Knowledge, compliance and barrier to compliance with standard precautions.	knowledge of SP was low; only 37.0% of HCWs knew that SP includes hand washing before and after any direct contact with the patient. 50% of respondents always protect themselves from blood and body fluids injections. About a quarter of the respondents do not recap needles after use. 48% of HCWs had regular training in SP<br>HCWs were thought that wearing PPEs—such as gloves, aprons, gowns and goggles—might cause patients to panic sometimes (63.0%) and complying with SP sometimes interferes with the ability to provide care (38.0%). Sometimes, because of the demands of patient care, HCWs do not have enough time to comply with the rigours of SP (44.0%) and sometimes PPEs are not available.
[36] Ider et al., 2012	Perceptions of healthcare professionals regarding the main challenges and barriers to effective hospital infection control	Qualitative study	Mongolia	87 participants (35 infection control professionals and 8 other health professionals)	Challenges and barriers to successful implementation of infection control programmes in Mongolia- 1) the formulation; and (2) the implementation of infection control policy.	Poor IC education of health professionals; limited laboratory capacity; inappropriate use of antibiotics; low compliance with hand hygiene; poor disinfection and sterilization; and poor implementation of occupational health programmes
[37] Al-Abhar, 2017	Knowledge and Practice of Biosafety Among Laboratory Staff Working in Clinical Laboratories	Cross sectional study	Yemen	362 participants	Knowledge level and practice of Laboratory Standard Precautions.	Of the private and public laboratory staff, 67% and 32% had received training on biosafety ($P < .001$), respectively. Overall, only 38% of respondents had good knowledge of LSP, 49% had fair knowledge, and 13% had poor knowledge. Only 32% of respondents had good practice of LSP, 59% had fair practice level, and 9% had poor practice.

[38] Chalya et al., 2016	Knowledge, practice and factors associated with poor compliance with universal precautions among healthcare workers	Cross sectional study	Tanzania	200 participants (34 laboratory staff)	The knowledge, practice and factors associated with poor compliance universal precautions among healthcare workers. Independent variables of interest were age, sex, job category, professional qualification, working place, working experience and previous training on universal precaution. The dependent (outcome) variable was compliance with universal precaution.	More than three quarters (82%) of participants had adequate knowledge of universal precautions. Out of 200 HCWs, 154 (77.0%) practiced universal precautions. training on universal precautions was significantly associated with good practice of universal precautions ($P < 0.001$). There was a strong correlation between knowledge and compliance (practice) with universal precautions ($r=0.76$). Lack of PPE, lack of knowledge and emergency situations accounted for the most frequently mentioned reasons for poor compliance.
[39] Fayaz et al., 2014	Knowledge and practice of universal precautions among health care workers	Cross sectional study	Afghanistan	300 participants (133 allied medical professionals)	Knowledge and practice of universal precautions.	Among the 300 respondents, the mean knowledge score was 5.2 with a standard deviation (SD) of 1.5. On the practice score, the mean was 8.7 (SD =2.2). A total of 90.6% and 70.8% of HCWs believed that UPs were necessary in contact with urine/feces and tears, respectively, although UPs are not necessary in these cases. On the other hand, 57.8% reported that they always recapped the needle after giving an injection, and 31.8% didnot always change gloves in between patients. There were no associations between the knowledge and self-reported practice of UPs.
[40] Kahhaleh and Jurjus, 2005	Adherence to universal precautions among laboratory personnel	Cross sectional study	Lebanon	290 participants	Vari-ables included the knowledge, attitudes and practices of laboratory technicians concer-ning blood-borne pathogens (e.g. HIV, HBV and HCV) and adherence to universal safety precautions in relation to experience, formal training and workplace setting among technicians dealing with blood and body-fluids, as well as laboratory directors.	.<br>Almost all the technicians knew that while working they should take protective measures by wearing laboratory gowns or gloves and that they should dispose of used needles and syringes in special containers. 45 (20.3%) had training on how to perform HIV testing. It was, however, observed that the technicians actually wore gloves in only 27 laboratories and laboratory coats in only 63.

[41] Jin et al., 2020	Perceived infection transmission routes, infection control practices, psychosocial changes, and management of COVID-19 infected healthcare workers	Cross sectional study	China	7 medical technicians	Perceived causes of infection, infection prevention, control knowledge and behaviour, psychological changes, symptoms and treatment were measured.	43 (41.8%) thought their infection was related to protective equipment, utilization of common equipment (masks and gloves). The main perceived mode of transmission was not maintaining protection when working at a close distance and having intimate contact with infected cases.
[42] Ngwa et al., 2018	Assessment of the knowledge, attitude and practice of health care workers in Fako Division on post exposure prophylaxis to blood borne viruses	Cross-sectional study	Cameroon	148 participants (68 laboratory staff)	Knowledge, attitude and practice of healthcare workers on post exposure prophylaxis and also determine the factors influencing reporting of occupational exposures among HCW	A high proportion of participants 58% had poor knowledge on Post Exposure Prophylaxis and 60.6% of participants proved to have a positive attitude towards post exposure prophylaxis. 50.9% (110/216) of all participants had at least one occupational exposure with a low uptake 19.1(21/110) of Post Exposure Prophylaxis recorded among participants who were exposed.
[43] Buxton et al., 2012	Prion disease risk perception	Cross-sectional study	Canada	426 medical laboratory workers.	knowledge, attitudes and reported behaviours of medical laboratory workers in relation to prion disease to understand their risk perception and the need for national laboratory guidelines on prion infection control.	18% believed they were at risk when processing these specimens. Less than one-third of those receiving specimens believed they were adequately trained. The mean (±SD) knowledge score was 9.25±4.5/24; individuals who had received training scored significantly higher than those who were untrained (P<0.01). 81% of respondents would be more comfortable processing specimens if national guidelines existed and were used in their laboratory.<br>There is a high perception of risk and few perceived benefits of processing prion-associated specimens. It is concerning that only onehalf of respondents who worked in laboratories reported that their protocols include standard precautions.
[44] Njagi et al., 2012	Knowledge, Attitude and Practice of Health-Care Waste Management and Associated Health Risks	Cross-sectional study	Kenya	At 599 participants at KNH and 261 at MTRH.	Identification of gaps in knowledge, attitude and practice in the management of health-care waste.	Most of them acquired knowledge on waste-management through on-job training from seminars and informally through organized talks at work- places. The hospital attendants had also an opportunity to acquire the knowledge through organized training at work places. The training improved the workers' compliance to hepatitis B vaccinations and use of

						personal protective equipment when handling health-care waste. handling medical waste.
[45] Woith et al., 2012	Barriers and Facilitators Affecting Tuberculosis Infection Control Practices of Russian Health Care Workers	Qualitative study	Russia	96 participants (12 laboratory staff)	How TB is transmitted and when a person is infectious; what IC methods were used and when these were used; and what barriers and motivators existed to use of infection control.	Barriers and motivators related to knowledge, attitudes and beliefs, and practices were identified. Three main barriers were a) knowledge deficits, including the belief that TB was transmitted by dust, linens, and eating utensils; b) negative attitudes related to the discomfort of respirators; and c) practices with respect to quality and care of respirators. Education and training, fear of infecting loved ones, and fear of punishment were the main motivators.
