# Supplementary figures and images for: Knowledge, attitude and practice of infection prevention and control precautions among laboratory staff: a mixed-methods systematic review
Source: Antimicrob Resist Infect Control. 2023 Jun 13;12:57. doi: 10.1186/s13756-023-01257-5 (PMC10262112; doi:10.1186/s13756-023-01257-5)

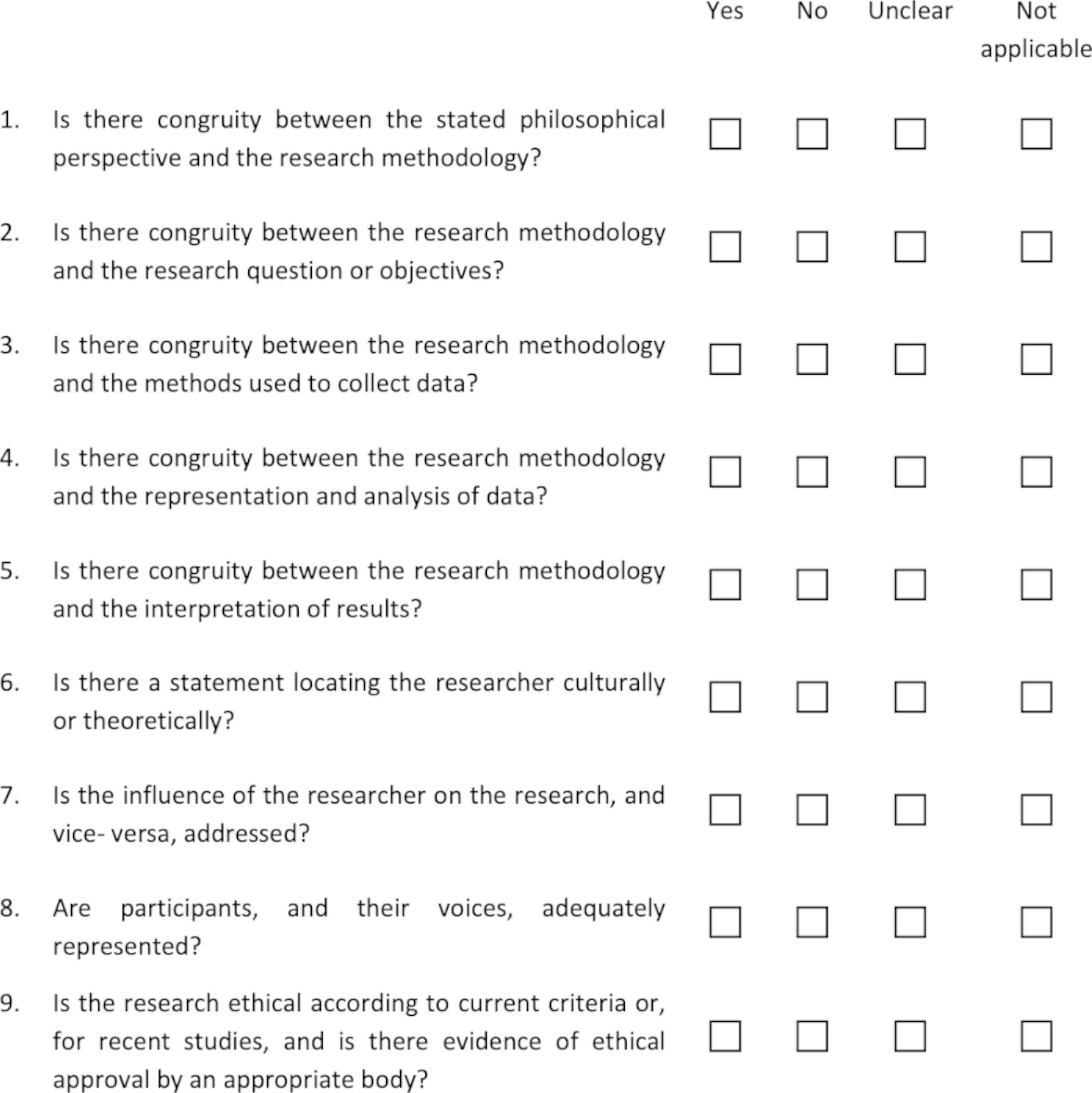

Supplement: Supplementary file 2 — Additional file 1: JBI Critical Appraisal Checklist for Cross-sectional Studies [file 13756_2023_1257_MOESM2_ESM.tif]

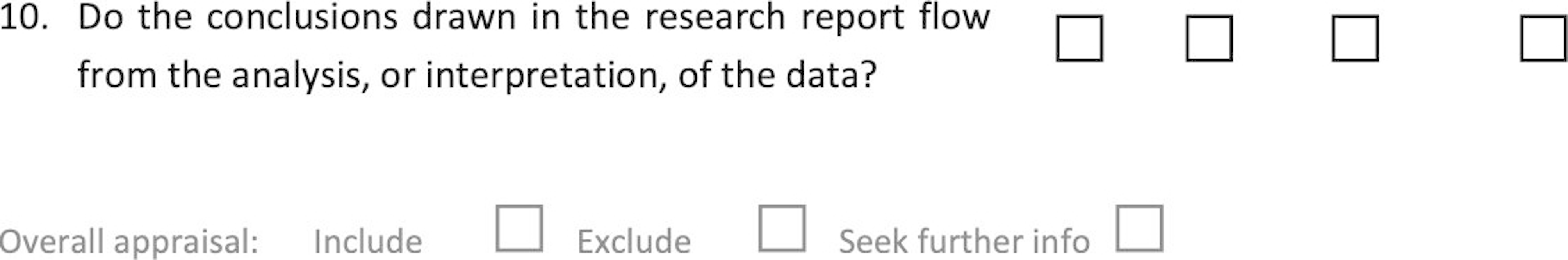

Supplement: Supplementary file 3 — Additional file 2: JBI Critical Appraisal Checklist for Qualitative Research [file 13756_2023_1257_MOESM3_ESM.tif]
